# Interaction of Some Commercial Teas with Some Carbohydrate Metabolizing Enzymes Linked with Type-2 Diabetes: A Dietary Intervention in the Prevention of Type-2 Diabetes

**DOI:** 10.1155/2014/534082

**Published:** 2014-01-16

**Authors:** Ganiyu Oboh, Omodesola O. Ogunruku, Funke O. Ogidiolu, Adedayo O. Ademiluyi, Bukola C. Adedayo, Ayokunle O. Ademosun

**Affiliations:** ^1^Functional Foods and Nutraceuticals Unit, Biochemistry Department, Federal University of Technology, PMB 704, Akure, Nigeria; ^2^Biochemistry Department, Obafemi Awolowo University, Ile-Ife, Nigeria

## Abstract

This study is aimed at assessing the inhibitory effect of teas on key enzymes (*α*-amylase and *α*-glucosidase) linked with type-2 diabetes and their antioxidant properties. Four samples of three brands were used; infusions of green tea (GT), 2 brands of black tea (BT), and a formulated herbal preparation for diabetes (ADT) (white tea, *Radix Puerariae*, *Radix ophiopogonis*, hawthorn berry, Chinese yam, and fragrant Solomon seal rhizome) were prepared and subsequently analyzed for their total phenol, ascorbic acid contents, antioxidant properties (2,2-Azizobis (3-Ethylbenzo-Thiazoline~6-sulfonate) “ABTS” scavenging ability and ferric reducing antioxidant property), and inhibition of pancreatic-*α*-amylase and intestinal-*α*-glucosidase *in vitro*. The study revealed that GT had the highest total phenol content, ascorbic acid content, ABTS∗ scavenging ability, and ferric reducing ability. Furthermore, all the teas inhibited Fe^2+^ and sodium nitroprusside induced lipid peroxidation in pancreas, with GT having the highest inhibitory effect. Conversely, there was no significant difference (*P* > 0.05) in the inhibitory effects of the teas on *α*-amylase and *α*-glucosidase. The antidiabetic property of the teas could be attributed to their inhibitory effect on carbohydrate hydrolyzing enzymes implicated in diabetes and their antioxidant activities.

## 1. Introduction

Diabetes mellitus (DM) is undoubtedly one of the most challenging health problems in the 21st century; statistics show that at 2011, 366 million people are suffering from DM, a figure expected to increase to 552 million people by 2030 [1]. DM is characterized by hyperglycemia associated with abnormal metabolism of carbohydrates, fats, and proteins resulting from endocrine defects in insulin action, secretion, or both [2]. The inhibition of alpha-amylase and alpha-glucosidase, carbohydrate hydrolyzing enzymes, can significantly reduce postprandial hyperglycemia and are thus considered an important therapeutic strategy in the management of blood glucose level in type-2 diabetes [3]. Inhibition of these enzymes delays the breakdown of polysaccharides and glucose absorption, thereby reducing the amount of glucose in the blood [4]. In recent times, there has been a growing interest in medicinal plants and functional foods and their disease modulatory effects.

Tea (*Camellia sinensis*) is the most consumed beverage in the world next to water, its consumption far exceeding beer, wine, and soft drink [5]. Teas are differentiated based on the manufacturing (fermentation) process: green tea (GT)-unfermented and black tea (BT)-fermented [5, 6]. There is however a third kind mostly popular in Asia commonly referred to as white tea; it shares similar processing to green tea but differs only in the stage of maturity in which the tea leaves are picked/harvested. Commercially grown teas are hybrids of two distinct types, the Assam type (var. *assamica*) and the China type (var. *sinensis*) [6]. GT from the genus of *Camellia* are rich sources of polyphenols particularly flavonoids of the subclass catechins and its derivatives called tea catechins or flavan-3-ols including catechin, epicatechin, epigallocatechin, epicatechin gallate, epigallocatechin gallate, and gallocatechin gallate. The fermentation process that produces BT results in the oxidation of the catechins by polyphenol oxidase into polymeric compounds, thearubigins, and theaflavins [5, 7, 8]. Several medicinal properties such as anticancer [8], hypocholesterolemic effects [9], antibacterial [10, 11], and antioxidant effects [12] that have been associated with tea are attributed to its rich polyphenol content. Several lines of study have shown an inverse relationship between tea consumption and hyperglycemia [13, 14], incidence of type-2 diabetes [15]. As a result, in this study the hypothesis that the molecular mechanisms by which teas from *Camellia sinensis* exert the observed antidiabetic effects that was through inhibition of carbohydrate metabolizing enzymes implicated in diabetes pathology was tested.

## 2. Materials and Methods

### 2.1. Sample Collection

Four different commercial samples of bagged tea leaves of three brands were purchased from supermarkets in Akure metropolis. These included green tea (GT), two black teas (BT1 and BT2), and a formulated herbal preparation for diabetes (ADT) composed of white tea (63.4%), *Radix puerariae* (8%), *Radix ophiopogonis* (6.2%), hawthorn berry (10%), Chinese yam (6.2%), and fragrant Solomon seal rhizome (6.2%).

### 2.2. Sample Preparation

1 g of each sample was extracted in 100 mL of hot water for about five minutes after which they were filtered. The filtrates were kept in −20°C until usage for subsequent analysis.

### 2.3. Determination of Vitamin C

Vitamin C content of the samples was determined using the method of Benderitter et al. [16]. Briefly, 75 *μ*L DNPH (2 g dinitrophenyl hydrazine, 230 mg thiourea, and CuSO_4_·5H_2_O in 100 mL of H_2_SO_4_) was added to 500 *μ*L reaction mixture (300 *μ*L of appropriate dilution of the extracts with 100 *μ*L of 13.3% trichloroacetic acid (TCA)) and water. The reaction mixture was subsequently incubated for 3 hours at 37°C, then 0.5 mL of 65% H_2_SO_4_ (v/v) was added to the medium, and the absorbance was measured at 520 nm using spectrophotometer. The vitamin C content of the extracts was subsequently calculated using ascorbic acid as standard.

### 2.4. Determination of Total Phenol Content

The total phenol content of the samples was determined according to the method of Singleton et al. [17]. Briefly, appropriate dilutions of tea extracts were oxidized with 2.5 mL 10% Folin-Ciocalteau' s reagent (v/v) and neutralized by 2.0 mL of 7.5% sodium carbonate. The reaction mixture was incubated for 40 minutes at 45°C and the absorbance was measured at 765 nm using a spectrophotometer. The total phenol contents were subsequently calculated and represented as gallic acid equivalents (GAE).

### 2.5. Total Antioxidant Capacity

The ABTS scavenging ability of the extracts was determined according to the method described by Re et al. [18]. ABTS radicals were generated by reacting aqueous solution of ABTS (7 mM) with K_2_S_2_O_8_ (final concentration of 2.45 mM) in the dark for 16 hours and adjusting the absorbance at 734 nm to 0.700. 200 *μ*L of the extract was added to 2.0 mL ABTS solution and the absorbance was measured at 734 nm after 15 minutes. The trolox equivalent antioxidant capacity was subsequently calculated.

### 2.6. Determination of Reducing Property

The Reducing property of the extract was determined by assessing the ability of the extracts to reduce FeCl_3_ solution as described by [19]. Briefly, appropriate dilutions of the extracts (2.5 mL) were mixed with 2.5 mL 200 mM sodium phosphate buffer (pH 6.6) and 2.5 mL of 1% potassium ferricyanide. The mixture was incubated at 50°C for 20 minutes. Thereafter, 2.5 mL 10% trichloroacetic acid was added and subsequently centrifuged at 650 rpm for 10 minutes. 5 mL of the resulting supernatant was mixed with equal volume of water and 1 mL of 0.1% ferric chloride. The absorbance was taken at 700 nm against a reagent blank.

### 2.7. Lipid Peroxidation Assay

#### 2.7.1. Tissue Preparation

The pancreas of the rat was quickly removed, placed on ice, and weighed. This tissue was subsequently homogenized in cold saline (150 mM) (1 : 10 w/v) using mortar and pestle. The homogenate was centrifuged for 10 minutes at about 2000 rpm to yield a pellet that was discarded and a low-speed supernatant (S1) was kept for lipid peroxidation assay [20].

#### 2.7.2. Lipid Peroxidation and Thiobarbituric Acid Reactions

The lipid peroxidation assay was carried out using the method of Pulido et al. [19] as modified by Puntel et al. [21] with two prooxidants (Fe^2+^ and sodium nitroprusside). Briefly, 1 mL of the S1 fraction was mixed with a reaction mixture containing 300 *μ*L and 10 mM Tris-HCl (pH 7.4), and the volume was made up to 3 mL with distilled water, then 300 *μ*L of the extract was added before incubation at 37°C for 1 hour. The colour reaction was developed by adding 3 mL of 8.1% SDS (sodium duodecyl sulphate) to the reaction mixture containing S1; this was subsequently followed by the addition of 5 mL acetic acid solution (pH 3.4) and 5 mL 0.6% TBA (thiobarbituric acid). This mixture was then incubated at 100°C for 1 hour. Thiobarbituric acid reactive species (TBARS) produced were measured at 534 nm and the absorbance was compared with that of standard curve using malondialdehyde (MDA).

### 2.8. Enzyme Inhibition Assays

#### 2.8.1. *α*-Amylase Inhibition Assay

Appropriate dilution of the tea extracts (500 *μ*L) and 500 *μ*L of 0.02 M sodium phosphate buffer (pH 6.9 with 0.006 M NaCl) containing Hog pancreatic *α*-amylase (EC 3.2.1.1) (0.5 mg/mL) were incubated at 25°C for 10 minutes. Thereafter, 50 *μ*L of 1% starch solution in 0.02 M sodium phosphate buffer (pH 6.9 with 0.006 M NaCl) was added to each tube. The reaction mixture was incubated at 25°C for 10 minutes and stopped with 1 mL of dinitrosalicylic acid (DNSA) colour reagent. Thereafter, the mixture was incubated in a boiling water bath for 5 minutes and cooled to room temperature. The reaction mixture was then diluted by adding 10 mL of distilled water, and absorbance measured at 540 nm.

#### 2.8.2. *α*-Glucosidase Inhibition Assay

Appropriate dilution of the extract (50 *μ*L) and 100 *μ*L of *α*-glucosidase solution (1 U/mL) in 0.1 M phosphate buffer (pH 6.9) was incubated at 37°C for 10 minutes. Then 40 *μ*l of 5 mM p-nitrophenyl-*α*-D-glucosidase solution in 0.1 M phosphate buffer (pH 6.9) was added. The mixture was incubated at 37°C for 10 minutes before reading the absorbance at 405 nm in the spectrophotometer. The *α*-glucosidase inhibitory activity was expressed as percentage inhibition.

### 2.9. Data Analysis

The results of triplicate studies were pooled and expressed as mean ± standard error of mean (SEM). A one-way analysis of variance was carried out. Significance was accepted at *P* > 0.05. EC_50_ was further determined by nonlinear regression analysis.

## 3. Results

**Figure 1:**
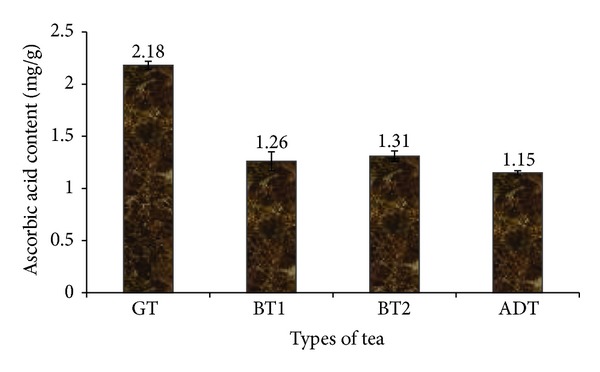
Ascorbic acid content of some commercially available tea. GT: Green tea; BT: Black tea; ADT: antidiabetes tea.

**Figure 2:**
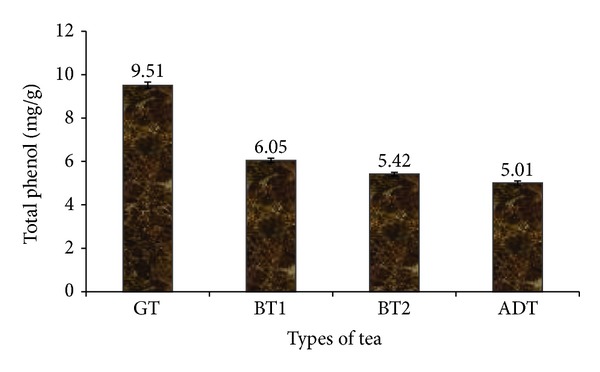
Total phenol content of some commercially available tea. GT: Green tea; BT: Black tea; ADT: antidiabetes tea.

In this study, the ascorbic acid contents of the tea brands as presented in Figure 1 revealed that green tea GT (2.18 ± 0.04 mg/g) was significantly (*P* < 0.05) higher in ascorbic acid than black teas (BT1: 1.26 ± 0.09 mg/g; BT2: 1.31 ± 0.05 mg/g) and antidiabetic tea (ADT: 1.15 ± 0.02 mg/g). Determined total phenol content (TPC) of infusions from some teas (Figure 2) shows that green tea (9.51 ± 1.55 mg/g) had a significantly (*P* < 0.05) higher phenolic content than fermented black teas (BT1: 6.05 ± 1.10 mg/g; BT2: 5.42 ± 0.98 mg/g) and the formulated antidiabetic tea (ADT: 5.01 ± 1.12 mg/g). However, there was no significant difference (*P* > 0.05) between the two brands of black teas (BT1 and BT2) and ADT.

The ABTS* scavenging ability of the tea represented as trolox equivalent antioxidant capacity (TEAC) are presented in Table 1. The results revealed that GT (5.54 ± 1.21) and ADT (4.70 ± 1.08) were significantly (*P* < 0.05) higher than the black teas (BT1: 1.80 ± 1.54; BT2: 2.80 ± 0.96). The fermented black teas also did not show any significant (*P* > 0.05) difference in their total antioxidant capacity when compared with each other. Furthermore, the ferric reducing antioxidant property was determined and is presented in Table 1 as ascorbic acid equivalents.

**Figure 3:**
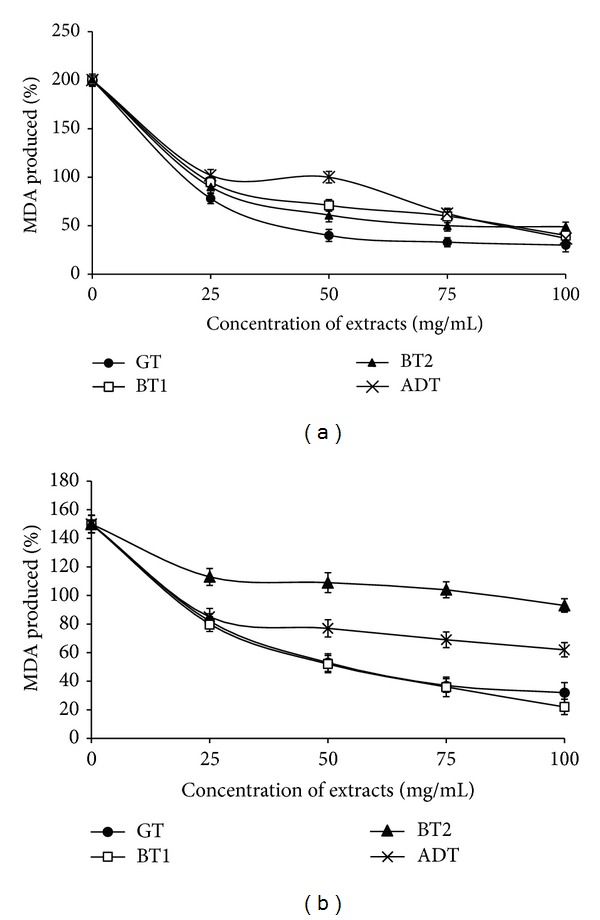
(a) Inhibition of Fe^2+^ induced lipid peroxidation in rat pancreas by aqueous extracts from some commercially available teas. GT: Green tea; BT: Black tea; ADT: antidiabetes tea. (b) Inhibition of sodium nitroprusside (SNP) induced lipid peroxidation in rat pancreas by tea infusion from some commercially available teas. GT: Green tea; BT: Black tea; ADT: antidiabetes tea.

We investigated further the antioxidant effect of the teas by interacting the tea infusions with isolated rat pancreas in the presence of Fe^2+^ and sodium nitroprusside (SNP) as prooxidants (Figures 3(a) and 3(b)). There was significant (*P* > 0.05) increase in the malondialdehyde (MDA) content of the pancreas after incubation with 25 *μ*M Fe^2+^ (200.0 ± 6.1%) and 5 mM SNP (150 ± 3.4%). However, the tea infusions caused a dose (25–100 mg/mL) dependent decrease in the MDA content of the pancreas in the presence of Fe^2+^ and SNP. The EC_50_ results show that GT (2.77 ± 0.86) was most effective against Fe^2+^ induced lipid peroxidation when compared with the other teas (BT1: 3.37 ± 1.09; BT2: 3.42 ± 0.95; ADT: 3.51 ± 1.34). Meanwhile, there was no significant (*P* < 0.05) difference in the ability of GT (3.41 ± 1.02) and BT1 (3.10 ± 1.27) to inhibit SNP induced lipid peroxidation. However, BT2 (7.62 ± 1.57) showed the least inhibitory effect.

**Figure 4:**
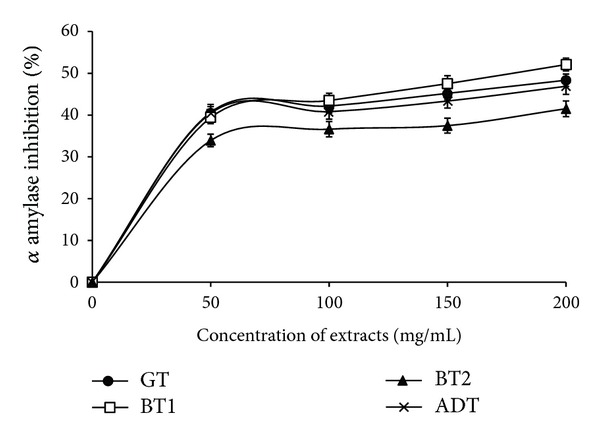
Inhibition of *α*-amylase by aqueous extracts of aqueous extracts from some commercially available teas. GT: Green tea; BT: Black tea; ADT: antidiabetes tea.

**Figure 5:**
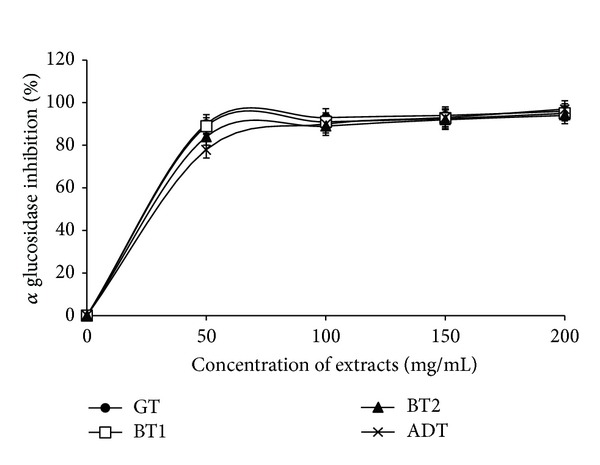
Inhibition of *α*-glucosidase by aqueous extracts from some commercially available teas. GT: Green tea; BT: Black tea; ADT: antidiabetes tea.

**Figure 6:**
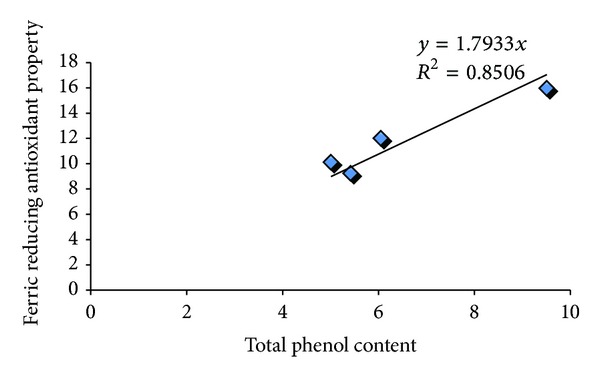
Relationship between the total phenol content and ferric reducing antioxidant properties of some commonly commercially available teas.

In a bid to further assess the antidiabetic potentials of the teas, the inhibitory activity of the tea infusions on *α*-amylase was determined (Figure 4). The teas showed a dose dependent inhibition of *α*-amylase (50–200 mg/mL). The EC_50_ results revealed that there was no significant difference in the ability of the tea infusions (BT1: 4.03 ± 1.21; GT: 4.36 ± 0.84; ADT: 4.62 ± 0.92) to inhibit *α*-amylase except in the case of BT2 (5.94 ± 1.42), where there was a significantly lower inhibitory activity. Similarly, the tea infusions inhibited *α*-glucosidase in a dose dependent manner (50–200 mg/mL) and the EC_50_ results also showed that there was no significant difference in the inhibitory activity of the teas (Figure 5).  Figure 6 showed the correlation between the total phenol content and ferric reducing antioxidant properties of the commercially available teas. The *R* ^2^ value was 0.8506, which showed a strong correlation.

## 4. Discussion

Evidences have accumulated in the last few years indicating that tea consumption is inversely correlated with the incidence of diabetes. We therefore sought to assess the ability of tea infusions to inhibit carbohydrate metabolizing enzymes and their antioxidant effects. The ascorbic acid content of the teas is lower than that reported for *Salvia officinalis* [22] but higher than that of *Capsicum pubescens* [23]. The difference in the ascorbic acid content of GT and BTs could have resulted from the fermentation processing of black teas. In a recent study by Moraes et al. [24] a decline in the ascorbic acid content of vegetables was also observed with different processing techniques. Furthermore, taking into consideration that the major constituent of the ADT used in this study is white tea; the significant difference in ascorbic acid between both could be a result of the maturity of the leaves. This is in agreement with studies by Lim and Quah [25], where increase in ascorbic acid was observed with maturity. All known physiological and biochemical actions of ascorbic acid is due to its actions as an electron donor whereby it loses two electrons sequentially resulting in the formation of relatively stable ascorbyl radical and dehydroascorbic acid, respectively. This known chemical property of vitamin C forms the basis for it free radical quenching ability [26].

The significant difference in total phenol contents between GT and BT agrees with earlier studies [27], which could be attributed to the fermentation process which black teas are subjected to. Kim et al. [5] reported a decrease of up to 37.2% in total soluble free phenolics of *Camellia sinensis* with increasing period in fermentation. TPC of the teas observed in this study is lower than previously reported values for green tea and black tea [6, 23] except for green tea, which was comparable to some other commercially available black teas in Argentina as reported by Anesini et al. [6]. This difference could be a result of the mode of extraction utilized. However, the TPC of the teas were also comparable to the total phenol content of *Salvia officinalis* [22], some citrus juices [28] and *Capsicum* spp. [29].

Reducing power is a novel antioxidation defense mechanism; the two mechanisms available to affect this property are by electron transfer and hydrogen atom transfer [30]. This is because the ferric-to-ferrous ion reduction occurs rapidly with all reductants with half reaction reduction potentials above that of Fe^3+^/Fe^2+^, the values in the Ferric reducing antioxidant property (FRAP) assay will express the corresponding concentration of electron-donating antioxidants [31]. The order of reducing power of the tea infusions is as follows GT > BT1 > ADT > BT2 (*P* < 0.05). These findings are similar to other studies, where GT had consistently shown a higher antioxidant activity than black teas [32]. These can be attributed to the significantly higher total phenol and ascorbic acid contents of green tea. There was strong correlation between the the total phenol content and ferric reducing antioxidant properties of the commercially available teas as shown by the *R* ^2^ value. This is similar to previous studies, where strong correlation between antioxidants properties of plant foods and their phenolic content have been established [32].

Lipid peroxidation has been implicated in various diseases resulting in free radical mediated release of MDA particularly from membrane lipids. As such, various kinds of antioxidants with different functions inhibit lipid peroxidation and the deleterious effects caused by the lipid peroxidation products [33]. The molecular mechanisms by which the Fe^2+^ could have resulted in the MDA increase could have been through its ability to participate in one-electron transfer reactions, whereby OH radicals are generated from H_2_O_2_ via the Fenton reaction. Iron has also been known to favor the propagation of lipid oxidation by decomposition of lipid peroxides resulting in peroxyl and alkoxyl radicals [22]. On the other hand, SNP toxicity is mediated through its release of cyanide and/or NO [34]. Although NO is considered to be cytoprotective at low concentrations, higher concentrations are considered toxic [35]. Its cytotoxicity is based on its ability to form peroxyl nitrite radicals in conjunction with other reactive oxygen species [36]. Furthermore, decomposition of SNP results in the release of iron that could further propagate the chain reaction of lipid peroxidation via the Fenton reaction [37].

The observed inhibitory effect of the teas on Fe^2+^ and SNP induced lipid peroxidation could have been a result of the teas being able to neutralize the OH and NO radicals produced by the prooxidants. Also there is the possibility that the tea extracts were able to chelate Fe^2+^ thereby attenuating its exacerbating effect as observed in the decrease in MDA content when incubated with the tea extracts. Plant polyphenols are known to exert antioxidant activity through the reduction/chelation of transition metals or scavenging of free radicals [38]. The higher inhibitory property of GT is worthnoting and could be attributed to the higher bioactivity of the tea catechins. It is noteworthy that the observed antioxidant activities of the teas at the concentrations used is similar to previous study [39].

Hyperglycemia is characterized by abnormal increases in postprandial blood glucose and is complicit in the etiology of type-2 diabetes [40, 41]. Increased activities of pancreatic *α*-amylase and intestinal *α*-glucosidase enzymes involved in starch hydrolysis have been reported in experimental diabetic animal models [42]. This justifies the pharmacological use of *α*-amylase and *α*-glucosidase inhibitors in treatment and management of type-2 diabetes. The use of phenolics particularly from dietary sources has been advocated as *α*-amylase and *α*-glucosidase inhibitors [43].

Furthermore, in a meta-analysis, [15] reported estimates indicating that individuals who drank more than 3 to 4 cups of tea per day had an approximate one-fifth lower risk of DM than those consuming no tea. Interestingly, several lines of study have revealed that GT can lower postprandial hyperglycemia and its associated complication [44]. The results from this study underscore the possible mechanism by which teas could have exerted their hypoglycemic effect and antidiabetic potentials.

## 5. Conclusion

It is possible to conclude from this study that green tea has higher antioxidant activity that could be attributed to its higher total phenol and ascorbic acid contents. However it was no more effective than the other teas in its antidiabetic potentials.

## Figures and Tables

**Table 1:** 2,2-Azizobis (3-ethylbenzothiazoline~6-sulfonate) “ABTS” scavenging ability and ferric reducing antioxidant property of some commercial teas.

Sample	ABTS (mmol·TEAC/100 g)	FRAP (AAE mg/g)
GT	5.54 ± 1.21^a^	15.98 ± 2.31^a^
BT1	1.80 ± 1.54^b^	12.01 ± 1.87^b^
BT2	2.80 ± 0.96^b^	9.26 ± 1.32^d^
ADT	4.70 ± 1.08^a^	10.12 ± 1.91^c^

GT: green tea; BT: black tea; ADT: antidiabetes tea. Values represent means of triplicate of duplicate experiments. Values with the same letter along the same column are not significantly different (*P* > 0.05).
